# Heat Transfer of Nanofluid in a Double Pipe Heat Exchanger

**DOI:** 10.1155/2014/736424

**Published:** 2014-11-10

**Authors:** Reza Aghayari, Heydar Maddah, Malihe Zarei, Mehdi Dehghani, Sahar Ghanbari Kaskari Mahalle

**Affiliations:** ^1^Department of Chemical Engineering, Shahrood Branch, Islamic Azad University, Shahrood 36199-43189, Iran; ^2^Department of Chemistry, Sciences Faculty, Arak Branch, Islamic Azad University, Arak, Iran; ^3^Department of Chemical Engineering, Damghan Branch, Islamic Azad University, Damghan 36716-39998, Iran; ^4^Department of Chemistry, Saveh Branch, Islamic Azad University, Saveh 39197-15179, Iran

## Abstract

This paper investigates the enhancement of heat transfer coefficient and Nusselt number of a nanofluid containing nanoparticles (*γ*-AL_2_O_3_) with a particle size of 20 nm and volume fraction of 0.1%–0.3% (V/V). Effects of temperature and concentration of nanoparticles on Nusselt number changes and heat transfer coefficient in a double pipe heat exchanger with counter turbulent flow are investigated. Comparison of experimental results with valid theoretical data based on semiempirical equations shows an acceptable agreement. Experimental results show a considerable increase in heat transfer coefficient and Nusselt number up to 19%–24%, respectively. Also, it has been observed that the heat transfer coefficient increases with the operating temperature and concentration of nanoparticles.

## 1. Introduction

The addition of solid particles into heat transfer media has long been known as one of the useful techniques for enhancing heat transfer, although a major consideration when using suspended millimeter- or micrometer-sized particles is that they have the potential to cause some severe problems, such as abrasion, clogging, high pressure drop, and sedimentation of particles. Compared to heat transfer enhancement through the use of suspended large particles, the use of nanoparticles in the fluids exhibited better properties relating to the heat transfer of fluid. This is because nanoparticles are usually used at very low concentrations and nanometer sizes. These properties prevent the sedimentation in the flow that may clog the channel. From these points of view, there have been some previous studies conducted on the heat transfer of nanoparticles in suspension. Since Choi wrote the first review article on nanofluids [1], Nguyen et al. [2] investigated the heat transfer coefficient and fluid flow characteristic of Al_2_O_3_ nanoparticles dispersed in water flowing through a liquid cooling system of microprocessors under turbulent flow condition. The results revealed that the nanofluid gave a higher heat transfer coefficient than the base liquid and the nanofluid with a 36 nm particle diameter gave higher heat transfer coefficient compared to the nanofluid with a 47 nm particle diameter. He et al. [3] reported an experimental study that investigated the heat transfer performance and flow characteristic of TiO_2_-distilled water nanofluids flowing through a vertical pipe in an upward direction under a constant heat flux boundary condition in both a laminar and a turbulent flow regime. Their results showed that at a given Reynolds number and particle size, the heat transfer coefficient is raised with increasing nanoparticle concentration in both laminar and turbulent flow regimes. Similarly, heat transfer coefficient was not sensitive to nanoparticle size at a given Reynolds number and particle size. Moreover, the results indicated that the pressure drop of the nanofluids was very close to that of the base fluid.

## 2. Experimental

### 2.1. Experimental Setup

**Figure 1:**
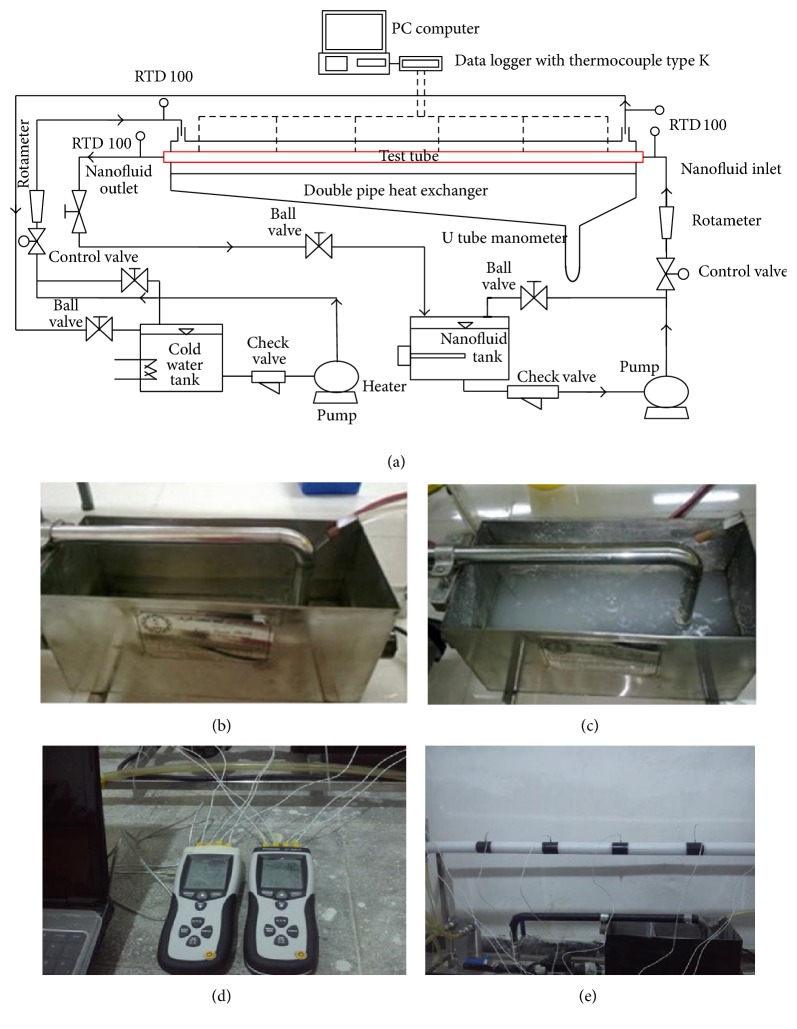
Experimental setup.

Experimental apparatus used in this study is depicted in Figure 1. The apparatus consists of a test section (heat exchanger), two tanks, two magnetic gear pumps, and a pump for transporting nanofluid as the hot fluid and the other for the cold water. The test section is a counter current double pipe heat exchanger with the length of 120 cm. In this exchanger, the nanofluid flows into the pipe and cold water in the annular space of the pipe. The inside pipe is made of a soft steel tube with the inner diameter of 6 mm, outer diameter of 8 mm, and thickness of 2 mm while the outside pipe is of steel tube with the inner diameter of 14 mm, outer diameter of 16 mm, and thickness of 2 mm. To reduce the heat loss along the axis, the top and bottom of the test section are insulated with the plastic tubes. To measure the inlet and outlet temperature of the nanofluid and cold water at the inlet and outlet of the test section, 4 RTD thermometers are used. It is necessary to measure the temperature at six stations altogether at the outer surface of the test section for finding out the average Nusselt number. All six evaluated temperature probes are connected to the data logger sets. The pressure drops across the test section are measured by using inclined U-tube manometers. The 15-liter tanks made of stainless steel are used for the storage of nanofluid and cold water. To maintain the temperature of the fluid, a cooling tank and a thermostat are used. An electric heater and a thermostat installed on it are used to maintain the temperature of the nanofluid. Measured Nusselt number error depends on the measurement of the temperature and the flows of the cold water and nanofluid. During the test, the wall temperature of the test section, the mass flow rate, and the inlet and outlet temperatures of the nanofluid and cold water are measured.

### 2.2. Nanofluid Preparation

The nanofluid used in the experiment was 99.0+% pure aluminum oxide predispersed in water, with an average particle size of 20 nm. The nanofluid was mixed with deionized water. To prepare experimental concentrations, nanofluids with less than 4% nanoparticles were found to be stable and the stability lasted over a week; no intermediate mixing was considered necessary (Table 1).

### 2.3. Data Processing

The experimental data were used to calculate overall heat transfer coefficient, convective heat transfer coefficient, and Nusselt number of nanofluids with various particle volume concentrations and Peclet numbers. For fluid flows in a concentric tube heat exchanger, the heat transfer rate of the hot fluid (nanofluid AL_2_O_3_) in the inner tube can be expressed as

(1) Qnano fluidhot fluid =mnano fluidhot fluid°Cpnano fluidhot fluidTout−Tin,

where *m*° is the mass flow rate of the nanofluid (hot fluid) and *T*_out_ and *T*_in_ are the outlet and inlet temperatures of the nanofluid (hot fluid), respectively.

The heat transfer of the cold fluid (water) for the outer tube is

(2) Qcold fluidwater =mcold fluidwater°Cpcold fluidwaterTin−Tout,

where *m*° is the mass flow rate of the water (cold fluid) and *T*_in_ and *T*_out_ are the inlet and outlet temperatures of the water (cold fluid), respectively.

The effective density of nanofluid is

(3) $$\begin{array}{c} \rho_{\text{nf}}=\left(1-\varphi_{V}\right)\rho_{f}+\varphi_{V}\rho_{p}. \end{array}$$

Subscripts *f*, *p*, and nf refer to the base fluid, the nanoparticles, and the nanofluid, respectively. *φ*_*V*_ is the nanoparticle volume concentration. *C*_*p*_nf__ is the effective specific heat of the nanofluid which can be calculated from Xuan and Roetzel relation [4]:

(4) $$\begin{array}{c} {\left(\rho C_{p}\right)}_{\text{nf}}=\left(1-\varphi_{V}\right){\left(\rho C_{p}\right)}_{f}+\varphi_{V}{\left(\rho C\right)}_{p}. \end{array}$$

The heat transfer coefficient of the test fluid, *h*_*i*_, can be calculated as follows [5]:

(5) $$\begin{array}{c} \frac{1}{U_{i}}=\frac{1}{h_{i}}+\frac{D_{i}\text{Ln}\left(D_{o}/D_{i}\right)}{{2k}_{w}}+\frac{D_{i}}{D_{o}}+\frac{1}{h_{o}}, \end{array}$$

where *D*_*i*_ and *D*_*o*_ are the inner and outer diameters of tubes, respectively, *U*_*i*_ is the overall heat transfer coefficient based on the inside tube area, *h*_*i*_ and *h*_*o*_ are the individual convective heat transfer coefficients of the fluids inside and outside the tubes, respectively, and *k*_*w*_ is the thermal conductivity of the tube wall. *U*_*i*_ is given by

(6) $$\begin{array}{c} Q=U_{i}A_{i}{\Delta T}_{\text{lm}}, \end{array}$$

where *A*_*i*_ = *πD*_*i*_ *L* and Δ*T*_lm_ is the logarithmic mean temperature difference. The outside heat transfer coefficient can be computed by Bell's procedure [6]. Nusselt number of nanofluids is defined as follows.

The convection heat transfer from the test section can be written by

(7) $$\begin{array}{c} Q_{\left(\text{convection}\right)}=h_{i}A_{i}\left(T_{w}^{\sim }-T_{b}\right),\\ T_{b}=\frac{T_{\text{out}\left(\text{nano fluid}\left(\text{hot fluid}\right)\right)}+T_{\text{in}\left(\text{nano fluid}\left(\text{hot fluid}\right)\right)}}{2},\\ \left(T_{w}^{\sim }=\sum \frac{T_{w}}{6}\right), \end{array}$$

where *T*_*w*_ is the local surface temperature at the outer wall of the inner tube. The average surface temperature *T*_*w*_^~^ is calculated from 6 points of *T*_*w*_ lined between the inlet and the exit of the test tube. The heat transfer coefficient *h*_*i*_ and the Nusselt number Nu are estimated as follows:

(8) hi=mnano fluidhot fluid°Cpnano fluidhot fluidTout−TinAiTw~−Tb,Nunf=hidiknf,

where the effective thermal conductivity (*k*_nf_) of the nanofluids can be evaluated by Maxwell's model that is given as follows [7]:

(9) $$\begin{array}{c} k_{\text{nf}}=k_{f}\frac{k_{p}{+2k}_{f}-2\varphi_{V}\left(k_{f}-k_{p}\right)}{k_{p}{+2k}_{f}+\varphi_{V}\left(k_{f}-k_{p}\right)}. \end{array}$$

Maxwell's formula shows that the effective thermal conductivity of nanofluids (*k*_nf_) relies on the thermal conductivity of spherical particles (*k*_*p*_), the thermal conductivity of base fluid (*k*_*f*_), and volume concentration of the solid particles (*φ*_*V*_).

## 3. Results and Discussion

**Figure 2:**
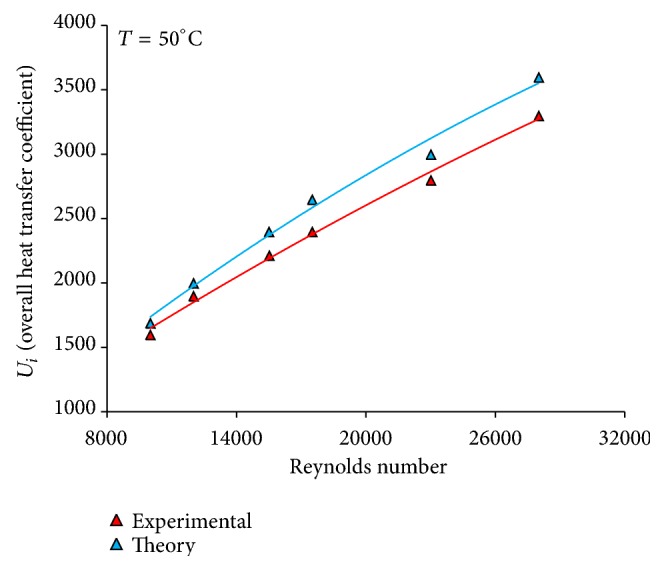
Comparison between the measured overall heat transfer coefficient and predicted values for distilled water.

To evaluate the accuracy of the measurements, experimental system was tested with distilled water before measuring the convective heat transfer of nanofluids.  Figure 2 shows the comparison between the measured overall heat transfer coefficient and prediction of (5) in which *h*_*i*_ is evaluated by Gnielinski correlation for turbulent flow through a tube [8]:

(10) Nu=0.012Re0.87−280Pr⁡0.4.

As shown in Figure 2, the good agreement exists between the experimental data and predicted values.

### 3.1. The Convective Heat Transfer of the Nanofluid

**Figure 3:**
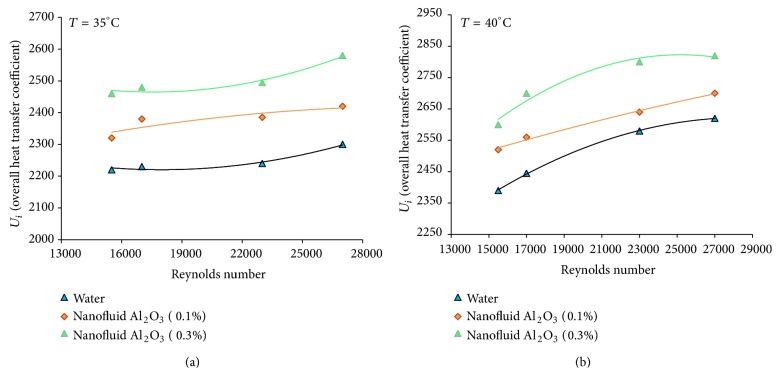
Overall heat transfer coefficient of *γ*-Al_2_O_3_/water nanofluid versus Reynolds number for various volume concentrations (*T* = 35°C, 40°C).

**Figure 4:**
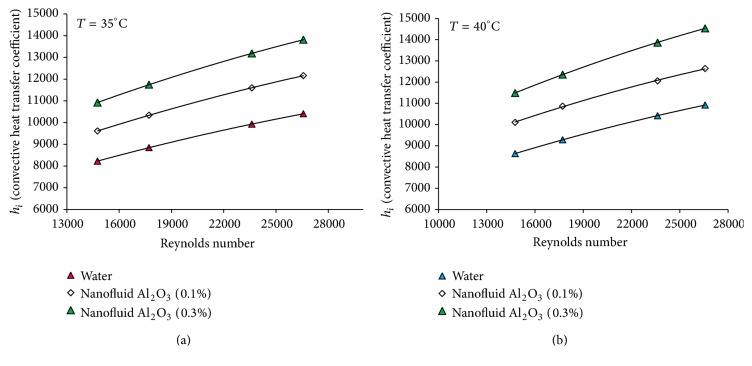
Convective heat transfer coefficient of *γ*-Al_2_O_3_/water nanofluid versus Reynolds number for different volume concentrations (*T* = 35°C, 40°C).

**Figure 5:**
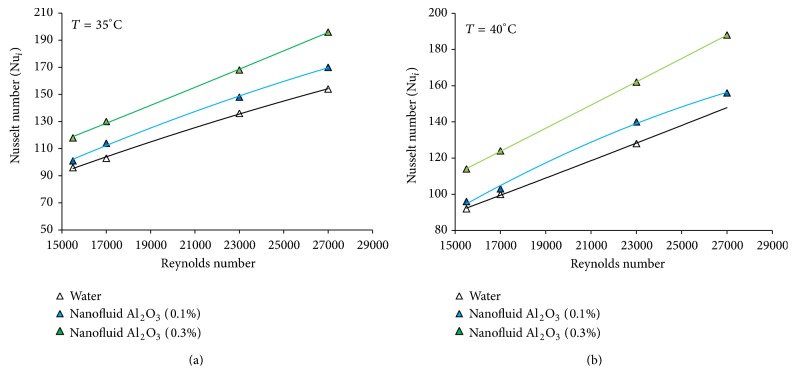
Nusselt number of *γ*-Al_2_O_3_/water nanofluid versus Reynolds number for different volume concentrations (35°C, 40°C).

Figure 3 shows the overall heat transfer coefficient of aluminum oxide nanofluid and water in terms of the Reynolds number at different volume concentrations show. The results show the increase of the overall heat transfer coefficient with the Reynolds number and temperature of the nanofluid. Compared to the base fluid, the heat transfer coefficient of aluminum oxide nanofluid increases with the increase of concentration in a fixed Reynolds number. The overall heat transfer coefficient is found to be the highest for aluminum oxide nanofluid at the concentration of 0.3 and a Reynolds number of about 27000, increasing up to 5 and 9.2% at the temperatures of 35 and 40°C compared to the base fluid. For water, this value is 4.6 and 6.82 percent for the temperatures of 35 to 40°C (Reynolds number of 27000 and concentration of 0.1). This increase in the convective heat transfer coefficient is also observed in Figure 4. For example, this value increases to 24.12 and 32.20% for the temperatures of 35–40°C compared to the base fluid (the concentration of 0.3 and Reynolds number of 27000). For water, this amount is 21.3 and 24.35 percent at the same Reynolds number and the concentration of 0.1. As seen in Figure 3, the overall heat transfer coefficient increases with the increase of Reynolds number. The possible reasons for this increase may be as follows:

1. a nanofluid with suspended nanoparticles which increases the thermal conductivity of the mixture,
2. high energy exchange process, which is resulted from the amorphous movement of the nanoparticles. Comparison of convective heat transfer coefficient between the nanofluid and the base fluid shows that this value is higher for the nanofluid at the same Reynolds number than the base fluid (Figure 4). This results in the increase of heat transfer efficiency caused by the increase of thermal conductivity, convective heat transfer, and the thinness of thermal boundary layer.  Figure 5 shows the effects of temperature and concentration of aluminum oxide nanofluid in terms of the Nusselt number at the temperatures of 35 and 40°C, respectively. As can be seen, Nusselt number of the nanofluid under the condition of same Reynolds number is greater than the base fluid. For example, this value is 19% for the nanofluid with a concentration of 0.3 at the temperature of 35°C compared to the base fluid (the Reynolds number of 26500). This amount is 25% at the temperature of 40°C. This increase can be attributed to the thermal conductivity. There are several mechanisms to increase the thermal conductivity of the nanofluid: the formation of the liquid layer on the surface of the nanoparticles, Brownian motion, classification of particles, the transmission of the phonons projectiles in the nanoparticles, and the increase of the thermal conductivity of fluids with the increase of the nanoparticles in the pipe wall. The increase in the thermal conductivity can increase the heat transfer coefficient in the thermal boundary layer near the tube wall. Temperature is one of the factors increasing the thermal conductivity of the nanofluid and thereby increasing the heat transfer coefficient and Nusselt number. Experimental results indicate that the effects of the nanoparticles on the thermal conductivity increase with the temperature. It is assumed that the main mechanism for the thermal conductivity of the nanofluid is the random motion of the nanoparticles. This pseudo-Brownian motion is a function of fluid temperature. Thus, the increase in the thermal conductivity is higher for smaller particles than for larger particles at the high temperatures. Brownian motion at low temperatures is of less importance and therefore the difference in the increase of the thermal conductivity between the smaller and larger particles is reduced.

### 3.2. Comparison between Experimental Results and Available Correlations

**Figure 6:**
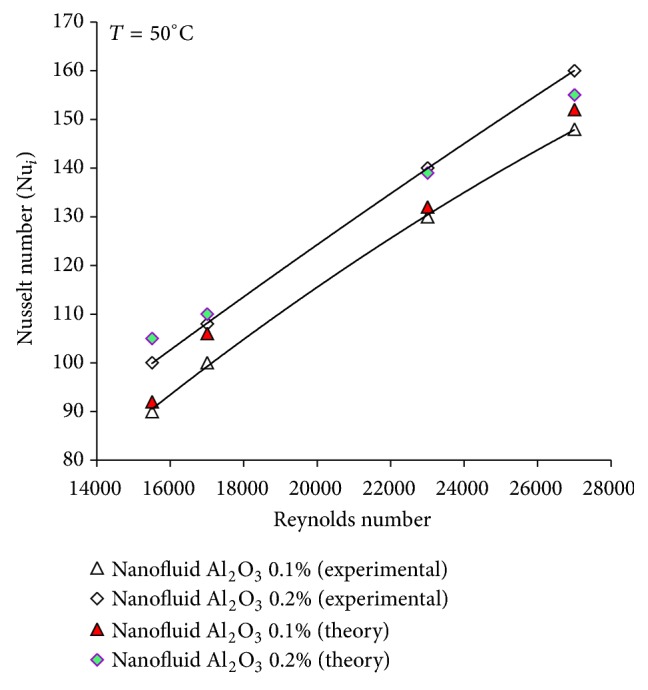
Comparison between the experimental results and calculated values from correlation 11 for *γ*-Al_2_O_3_/water nanofluids.

In Figure 6 the experimental results for the Nusselt number of *γ*-Al_2_O_3_/water nanofluid are compared with the prediction of Xuan and Li correlation. The correlation was provided by Xuan and Li for turbulent flow of nanofluid inside a tube [9]:

(11) Nunf=0.00591+7.6286φV0.6886Pep0.001Renf0.9238Pr⁡nf0.4.

As seen in Figure 6, there is an agreement between the experimental and calculated values for nanofluid. In the present study, aluminum oxide nanoparticles mixed with water to the volume percent of 0.1–0.3% (V/V) are used to investigate the effects of Reynolds number, the temperature of the flowing nanofluid, and the nanoparticle concentration on the heat transfer. Nusselt number increases with the Reynolds number. The obtained results are consistent with the results from the relationship between Xuan and Li [9]. The particle Peclet number, Reynolds number, and the Prandtl number for nanofluid are defined, respectively, as

(12) Pep=Vmdpαnf,Renf=VmDϑnf,Pr⁡nf=ϑnfαnf,

where the thermal diffusivity is given by

(13) $$\begin{array}{c} \alpha_{\text{nf}}=\frac{k_{\text{nf}}}{{\left(\rho C_{p}\right)}_{\text{nf}}}=\frac{k_{\text{nf}}}{{\left(1-\varphi_{f}\right)\left(\rho C_{p}\right)}_{f}+\varphi_{f}\left(\rho C_{p}\right)p}. \end{array}$$

## 4. Conclusion

With respect to utilizing nanoparticles in many processes, attention has been focused on the improvement of heat exchanger efficiency by adding solid particles to heat transfer fluids. Many researches have investigated the effect of nanoparticles on different process parameters like hydrodynamic and thermophysical properties. However, researches were seldom performed to evaluate the effect of turbulent nanofluid flow on heat transfer. This study investigated the heat transfer enhancement of the nanofluid containing aluminum oxide nanoparticles and water under the condition of turbulent flow in a double pipe heat exchanger. The heat transfer values were measured in the turbulent flow of a nanofluid containing 20 nm aluminum oxide suspended particles with the volume concentration of 0.1–0.3% (V/V) in water. Properties of nanofluid are good and there is plenty of fluid. Heat transfer coefficient and Nusselt number of the nanofluid increase from 15 to 20% compared to the base fluid according to the comparison on the basis of fixed Reynolds number. Experimental results showed the increase of the average heat transfer coefficient in the turbulent flow regime with the addition of the nanoparticles to the fluid. The obtained results are in agreement with the results from the relationship between Xuan and Li [9]. This increase in the heat transfer coefficient may be due to the high density of nanoparticles on the wall pipe and the migration of the particles. The extensive research is needed to understand the heat transfer characteristics of the nanofluid and to obtain the other relations.

## Figures and Tables

**Table 1:** Physical properties of the nanoparticles.

Types of nanoparticles	Nanosized particles	Special surface	Percent purity	Appearance	The apparent density	
γ-Al_2_O_3_	20 nm	>160 m^2^/g	+99	White powder	0.9 g/m^3^	

Certificate of analysis (content of elements)						
Co	M	N	Cl	V	Ca	Al_2_O_3_

≤2 ppm	≤3 ppm	≤70 ppm	≤315 ppm	≤7 ppm	≤25 ppm	≥99%

## Nomenclature

*A*: Heat transfer area (m^2^)
*C*_*p*_: Specific heat (kJ kg^−1^ °C^−1^)
*D*: Tube diameter (m)
*d*: Nanoparticle diameter (m)
*h*: Convective heat transfer coefficient (W m^−2^ °C^−1^)
*k*: Thermal conductivity (W m^−1^ °C^−1^)
*L*: Tube length (m)
*m*°: Mass flow rate (kg s^−1^)
Nu: Nusselt number (dimensionless)
Pe: Peclet number (dimensionless)
Pr: Prandtl number (dimensionless)
*Q*: Heat transfer rate (W)
Re: Reynolds number (dimensionless)
*T*: Temperature (°C)
*U*: Overall heat transfer coefficient (W m^−2^ °C^−1^)
*V*: Velocity (m^2^ s^−1^).

### Greek Symbols

Δ*T*_*Im*⁡_: Logarithmic mean temperature difference ( °C)
*α*: Thermal diffusivity (m^2^/s)
*ρ*: Density (kg m^−3^)
*ϑ*: Kinematic viscosity (m^2^/s)
*φ*_*V*_: Nanoparticle volume concentration (dimensionless).

### Subscripts

*f*: Fluid
*i*: Inside
in: Inlet
*m*: Mean
nf: Nanofluid
*o*: Outside
out: Outlet
*p*: Particles
*w*: Wall.
